# Fluctuation in body composition and urine specific gravity of Turkish wrestlers in a top-level official wrestling competition

**DOI:** 10.3389/fphys.2024.1516149

**Published:** 2025-01-27

**Authors:** Ersel Donmez, Ozkan Isik, Mario Baic

**Affiliations:** ^1^ Institute of Health Sciences, Balikesir University, Balikesir, Türkiye; ^2^ Faculty of Sports Sciences, Balikesir University, Balikesir, Türkiye; ^3^ Faculty of Kinesiology, University of Zagreb, Zagreb, Croatia

**Keywords:** combat sports, fat mass, hydration status, rapid weight loss, weight gain

## Abstract

**Purpose:**

This research aimed to examine the fluctuations in body composition and Urine-Specific Gravity (U_SG_) of elite wrestlers in a high-level official wrestling competition.

**Method:**

Thirty-one wrestlers in the Türkiye Senior Greco-Romen Wrestling national team participated in this research. Wrestlers were divided into weight loss and non-weight loss groups, and changes in their body composition and U_SG_ were measured at three different times (beginning of the camp, weigh-in, and before the competition). The duration between the beginning of the camp and the competition weigh-in time was considered as a dehydration process and the duration between the competition weigh-in time and before the competition was regarded as a rehydration process. In the data analysis, 3 × 2 repeated measures ANOVA was used.

**Results:**

It was determined that during the dehydration process of wrestlers who lost weight, there was a decrease of 4.02%, 2.50%, 14.62%, and 2.66% in body weight, FFM, FM, and TBW, whereas, during the rehydration process, there was a gain of 1.85%, 1.77%, 2.63%, and 1.87%, respectively. In addition, it was determined that wrestlers who lost weight had a 0.87% increase in U_SG_ during the dehydration process and a 0.41% decrease in the rehydration process. The results show that wrestlers cannot regain body weight and FM lost in the hydration fluctuation during a competition, but they can regain FFM, TBW, and U_SG_. However, although it was determined that the wrestlers were statistically able to regain FFM, TBW, and U_SG_, they could not return to the levels at the beginning of the camp.

**Conclusion:**

It was determined that before a high-level official wrestling competition, wrestlers still preferred weight loss practices and it was determined that they were exposed to fluctuations in their body composition and U_SG_. It is thought that this result may negatively affect the wrestlers’ health and competition performance during a high-level competition.

## 1 Introduction

Dehydration is the process of losing water from the body and rehydration is the process of regaining lost body water. Euhydration is the state of being in water balance. However, while the dictionary definition is easy, the physiological definition is not so easy. However, euhydration is not a static state. Rather it is a dynamic state of constantly losing water from the body and there may be a time lag before we can replace it, or we may take in a little extra and then lose it later (through urine or sweat) (Shirreffs, 2003). Therefore, being in euhydration is an important process for athletes to achieve athletic performance.

Especially in combat sports, athletes think that they gain an advantage by competing against opponents smaller or weaker than themselves (Ranisavljev et al., 2022; Rossi et al., 2021; Yalçın et al., 2019). For this reason, athletes generally prefer losing weight before weigh-in and regaining the weight lost after the weigh-in. This process causes irregularities and fluctuations in the hydration status of athletes. Of course, the physiological and psychological effects of these fluctuations are inevitable. Although many researchers have well-documented the physiological (Lakicevic et al., 2021; Lukic-Sarkanovic et al., 2024; Roklicer et al., 2020; Slačanac et al., 2021; Yagmur et al., 2019a; Yildirim, 2015) and psychological effects (Escobar-Molina et al., 2015; Isik and Dogan, 2017; Karninčić et al., 2016; Koca et al., 2023) of weight loss on the human body and international federations have tried to take many preventions regarding weight loss related to athlete health, athletes continue to perform these practices. For example, according to the 2014 Wrestling competition rules of United World Wrestling (UWW), the weigh-in was held the day before the competition (International Wrestling Rules, 2014) and the athletes believed that ∼17 h was sufficient for recovery. To prevent Rapid Weight Loss (RWL) practices, UWW has started to hold the weigh-in on the morning of the competition and ∼2 h before the competition for the last 10 years (International Wrestling Rules, 2018). In another example, the International Judo Federation holds the weigh-in the day before the competition but reweighs the athletes selected as a result of the draw on the morning of the competition, and athletes weighing 5% more than their competition weight are disqualified (International Judo Federation, 2024).

In recent years, researchers have demonstrated the effects of dehydration with many methods such as salivary flow rate (Cheuvront et al., 2010), hematological indicators (Dorwart and Chalmers, 1975; Yagmur et al., 2019b), cardiovascular indicators (Milovančev et al., 2023), monitoring of body weight decreases (Kavouras, 2002; Karninčić et al., 2013), changes in body composition [Fat Mass (FM), Fat-Free Mass (FFM), and Total Body Water (TBW)] using a bioelectrical impedance analyzer (BIA) (Isik and Dogan, 2017), and Urine-specific gravity (U_SG_) (Demirkan et al., 2010; Guder, 2020). Although BIA is a potential technique for assessing hydration status, it can be used as a quantitative diagnostic tool to investigate body water changes, but it is insufficient to determine dehydration alone and must be supported by another dehydration indicator (Kavouras, 2002). The fastest, most accurate, and most practical methods for determining dehydration are non-invasive methods such as monitoring of body weight reductions, BIA, and determination of U_SG_.

Wrestlers commonly use weight loss methods such as food and fluid restriction, repetitive intense exercise, jogging with a raincoat, and/or using the sauna (Wroble and Moxley, 1998). Of course, it has been reported that using these methods has physiological effects such as decreased glycogen storages, muscle cramps, injuries, heart palpitations, and increased body temperature, as well as psychological effects such as feeling extremely irritable and tired, stressed, and decreased desire to do sports (Franchini et al., 2012; Yarar et al., 2019). For these reasons, the NCAA has recommended that no more than 1.5% of body mass be lost per week. In addition, the NCAA has selected a U_SG_ value of 1.020 or less as a limit for euhydration status. Thus, it is predicted that the physiological and psychological effects of losing weight can be minimized (Utter, 2001).

Many studies in the literature on body composition and/or U_SG_ of wrestlers were generally related to pre-competition and/or dehydration. For this reason, this research was unique in that it simultaneously demonstrated the processes of dehydration and rehydration through both body composition and U_SG_ of wrestlers during an official competition. In this context, this research aimed to investigate the fluctuations in body composition and U_SG_ of elite wrestlers in a high-level official wrestling competition.

## 2 Methods

### 2.1 Calculation of sample size

To generalize the results of this study, the power of the sample size was calculated with G*Power software version 3.1.9.2. The total number required to find a statistically significant medium effect size (f = 0.50) in the fluctuations in body composition and U_SG_ of wrestlers who lost and did not lose weight was determined as 14 (7 people for each group) (α = 0.05; 1-β = 0.95). There were 21 wrestlers in the WL group and 10 wrestlers in the N-WL group, and this sample size shows that the results can be generalized.

### 2.2 Participants

This research was conducted with wrestlers who participated in the Yasar Dogu, Vehbi Emre and Hamit Kaplan Tournament, which was listed as a Ranking tournament by the UWW and was held in the February 2022 Turkish male Greco-Roman Wrestling National Team camp. Wrestlers included in the research did not have acute or chronic disease during the camp and did not use diuretics and/or laxatives. They were also warned not to consume beverages such as alcohol, energy drinks, and coffee the night before the measurements. At the beginning of this research, 38 wrestlers agreed to participate. However, 7 wrestlers were unable to urinate at the weigh-in time or refused to weigh themselves and urinate before starting the competition. As a result, the research was completed with 31 wrestlers ( $\bar{Χ}$ _Age_ = 24.87 ± 3.96 years; $\bar{Χ}$ _Height_ = 176.45 ± 8.80 cm) who volunteered to participate in the research in a 25-day national team camp and consisted of the sample of the research.

### 2.3 Experimental design

The age and height of the wrestlers were recorded as personal information and in order to determine their hydration status, questions such as “Do you perform weight loss?” For wrestlers who lost weight, “How many weeks before this competition do you start to lose weight?” and “Do you keep your body weight under control?” were asked. In addition, the wrestlers’ body weights were measured at the beginning of the camp and their official competition weights were asked. Thus, the wrestlers were divided into two groups as weight loss (n: 21) and non-weight (n: 10) groups and the body composition and U_SG_ of all wrestlers were measured at three different times: at the beginning of the camp, at the weigh-in and before the competition. The wrestlers who lost weight during the 23–24-day camp period between the beginning of the camp and the competition weigh-in time lost their weight as they wished without any intervention. The athletes’ competition weigh-ins were held between 8:00-9:00 on the day of the competition and the official competitions started at 10:30. The times between the start of the camp and the competition weigh-in were considered as dehydration for the wrestlers, and the times between the competition weigh-in and before the competition were considered as rehydration.

#### 2.3.1 Measurement of body composition

Determination of body composition is an important way to assess human health. BIA is a non-invasive and low-cost approach, widely used in the evaluation of body components and clinical status. In this research, the body compositions of wrestlers were measured with TANITA BC-418 (Tanita Corp., Tokyo, Japan) BIA in athletic mode, with a 250-gr. tare, barefoot, and in an official wrestling swimsuit at 3 different times (beginning of the camp, weigh-in, and pre-competition). FM, FFM, and TBW were measured separately for all body compositions of the wrestlers at each measurement time and recorded in kg.

#### 2.3.2 Measurement of U_SG_

The determination of U_SG_ is an important criterion for evaluating fluid balance and hydration status in the human body. This process involves comparing the density of urine with the density of water. In this research, urine samples collected in urine containers at 3 different times (beginning of the camp, weigh-in, and before the competition) were analyzed separately for each measurement time using a digital pen refractometer (PEN-PRO; Atago Co., Ltd., Tokyo, Japan) and the results were recorded in g*mL^−1^.

### 2.4 Ethical approval

All national team wrestlers were informed about the research procedures and the research’s aims, and written informed consent was obtained from the wrestlers who volunteered to participate in this research before participating in this research. In addition, research approval was obtained from the Turkish Wrestling Federation with protocol number E-56452965-125.99-1833853 and by the Balıkesir University Non-Invasive Research Ethics Committee with protocol number 2022/21. The research was conducted following the guidelines of the revised Declaration of Helsinki.

### 2.5 Statistical analysis

IBM SPSS Statistics 24 software was used in the analysis of the obtained data. Descriptive statistics of the obtained data were given as percentage, frequency, mean, and standard deviation. Two-way ANOVA (3 times × 2 groups) was used in repeated measurements to determine the difference between the measurement times of the weight loss and non-weight loss groups. The LSD *post hoc* test was used to determine the source of the difference between the measurement times. In addition, the percentage changes in the fluctuations in the body weights of the wrestlers were calculated with the formula “%Δ = [(Pre-Test–Post-Test)/Pre-Test] * 100” (Isik and Dogan, 2018; Isik et al., 2018). The confidence interval was chosen as 95%, and significance was considered as *p* < 0.05 and *p* < 0.01.

## 3 Results

When Table 1 was examined, it was determined that 67.7% of the wrestlers performed weight loss before the competition. Moreover, it was determined that 32.3% of the wrestlers started weight loss practice 1 week before the competition, 32.3% started 2 weeks before the competition, and 25.8% started 3 weeks before the competition. Additionally, it was determined that 81% of the wrestlers who performed weight loss and 50% of the wrestlers who did not perform weight loss kept their body weight under control in order not to gain body weight.

**TABLE 1:** Hydration status of Wrestlers.

Do you perform weight loss?											
Yes								No			
f				%				f		%	
21				67.7				10		32.3	
How many weeks before this competition do you start to lose weight?											
1 week			2 weeks			3 weeks					
f	%		f		%	f	%				
8	32.3		8		32.3	5	25.8				
Do you keep your body weight under control?								Do you keep your body weight under control?			
Yes				No				Yes		No	
f		%		f		%		f	%	f	%
17		81		4		19		5	50	5	50

When the body weight changes of the wrestlers were examined, it was determined that there was a statistically significant difference between the weight loss and non-weight loss groups (F = 4.740; *p* < 0.05). Moreover, when examined in terms of measurement times, the body weights of the wrestlers beginning the camp were found to be statistically different compared to the weigh-in and before the competition (F = 18.117; *p* < 0.01). In addition, the interaction between wrestler groups and measurement times was found to be statistically significant (F = 13.016; *p* < 0.01). According to this result, it was observed that the wrestlers who lost weight were exposed to a 4.02% body weight loss during the dehydration process and gained only 2.02% during the rehydration process. This result shows that wrestlers cannot regain their lost body weight before a high-level competition and were exposed to a statistical fluctuation in their body weight (Table 2).

**TABLE 2:** Comparison of fluctuations in wrestlers’ body weight in a high-level competition.

Variables	Groups/Times	n	Beginning of the camp		Weigh-in		Before the competition		Total	F	p
Body Weight (kg)	WL	21	81.55 ± 18.52		78.27 ± 18.17		79.72 ± 18.36		79.85 ± 18.35	4.740	0.038*
			Dehydration %Δ = ‒4.02			Rehydration %Δ = 2.02					
	N-WL	10	96.21 ± 21.06		95.97 ± 21.34		95.58 ± 20.83		95.92 ± 21.08		
			Dehydration %Δ = ‒0.25			Rehydration %Δ = ‒0.41					
	Total	31	88.88 ± 19.79^a^		87.12 ± 19.76^b^		87.65 ± 19.60^b^			Interaction F = 13.016; p < 0.001**	
	F = 18.117; p < 0.001**										

*p < 0.05; **p < 0.01; ab: Statistical differences between groups were shown with different letters.

When the changes in the wrestlers’ body FFM were examined, it was determined that there was a statistically significant difference between the weight loss and non-weight loss groups (F = 5.781; *p* < 0.05). Moreover, when the measurement times were examined, it was found that the wrestlers’ FFM averages at the weigh-in were statistically different from the averages at the beginning of the camp and before the competition (F = 6.241; *p* < 0.01). In addition, the interaction between wrestler groups and measurement times was found to be statistically significant (F = 4.294; *p* < 0.05). According to this result, it was observed that the wrestlers who performed weight loss were exposed to a 2.50% decrease in FFM during the dehydration process and gained 1.77% during the rehydration process. This result shows that wrestlers regain lost body FFM before a high-level competition (Table 3).

**TABLE 3:** Comparison of fluctuations in wrestlers’ FFM in a high-level competition.

Variables	Groups/Times	n	Beginning of the camp		Weigh-in		Before the competition		Total	F	p
FFM (kg)	WL	21	71.29 ± 11.43		69.51 ± 11.09		70.74 ± 11.21		70.52 ± 11.24	5.781	0.023*
			Dehydration %Δ = ‒2.50			Rehydration %Δ = 1.77					
	N-WL	10	81.70 ± 13.62		81.58 ± 14.17		81.86 ± 14.08		81.71 ± 13.96		
			Dehydration %Δ = ‒0.12			Rehydration %Δ = ‒0.34					
	Total	31	76.50 ± 12.53^a^		75.55 ± 12.63^b^		76.30 ± 12.65^a^			Interaction F = 4.294; p < 0.025*	
	F = 6.241; p < 0.006**										

*p < 0.05; **p < 0.01; ab: Statistical differences between groups were shown with different letters.

When the changes in body FM of wrestlers were examined, it was found that there was no statistically significant difference between the weight loss and non-weight loss groups (F = 2.571; *p* > 0.05). Moreover, when examined in terms of measurement times, it was found that the wrestlers’ FM averages at the beginning of the camp were statistically different from the averages at the weigh-in and before the competition (F = 7.213; *p* < 0.01). In addition, it was determined that the interaction between the wrestler groups and the measurement times was not statistically significant (F = 3.074; *p* > 0.05). According to this result, it was observed that the wrestlers who performed weight loss were exposed to a 14.62% decrease in FM during the dehydration process and gained 2.64% during the rehydration process. This result shows that wrestlers cannot regain their lost body FM before a high-level competition and were exposed to a statistical fluctuation in their body FM (Table 4).

**TABLE 4:** Comparison of fluctuations in wrestlers’ FM in a high-level competition.

Variables	Groups/Times	n	Beginning of the camp		Weigh-in		Before the competition		Total	F	p
FM (kg)	WL	21	10.26 ± 7.97		8.76 ± 7.82		8.99 ± 7.97		9.33 ± 7.92	2.571	0.120
			Dehydration %Δ = ‒14.62			Rehydration %Δ = 2.64					
	N-WL	10	14.52 ± 8.37		14.41 ± 8.15		13.74 ± 7.83		14.22 ± 8.12		
			Dehydration %Δ = ‒0.76			Rehydration %Δ = ‒4.64					
	Total	31	12.39 ± 8.17^a^		11.58 ± 7.99^b^		11.36 ± 7.90^b^			Interaction F = 3.074; p < 0.066	
	F = 7.213; p < 0.003**										

**p < 0.01; ab: Statistical differences between groups were shown with different letters.

When the changes in the TBW of the wrestlers were examined, it was determined that there was a statistically significant difference between the weight loss and non-weight loss groups (F = 5.787; *p* < 0.05). Moreover, when examined in terms of measurement times, it was found that the TBW averages of the wrestlers at the weigh-in were statistically different compared to the averages beginning the camp and before the competition (F = 6.864; *p* < 0.01). In addition, the interaction between wrestler groups and measurement times was found to be statistically significant (F = 4.888; *p* < 0.05). According to this result, it was observed that the wrestlers who performed weight loss were exposed to a 2.66% decrease in TBW during the dehydration process and regained 1.87% during the rehydration process. This result shows that elite wrestlers regain the TBW lost before a high-level competition (Table 5).

**TABLE 5:** Comparison of fluctuations in wrestlers’ TBW in a high-level competition.

Variables	Groups/Times	n	Beginning of the camp		Weigh-in		Before the competition		Total	F	p
TBW (kg)	WL	21	52.20 ± 8.37		50.81 ± 8.20		51.76 ± 8.24		51.59 ± 8.27	5.787	0.023*
			Dehydration %Δ = ‒2.66			Rehydration %Δ = 1.87					
	N-WL	10	59.79 ± 9.98		59.71 ± 10.37		59.93 ± 10.26		59.81 ± 10.20		
			Dehydration %Δ = ‒0.13			Rehydration %Δ = 1.34					
	Total	31	55.99 ± 9.18^a^		55.26 ± 9.29^b^		55.85 ± 9.25^a^			Interaction F = 4.888; p < 0.016*	
	F = 6.864; p < 0.004**										

*p < 0.05; **p < 0.01; ab: Statistical differences between groups were shown with different letters.

When the changes in wrestlers’ U_SG_ were examined, it was determined that there was no statistically significant difference between the weight loss and non-weight loss groups (F = 3.147; *p* > 0.05). Moreover, when the measurement times were examined, it was found that the average U_SG_ of the wrestlers at the weigh-in was statistically different compared to the average beginning the camp and before the competition (F = 7.531; *p* < 0.01). In addition, the interaction between wrestler groups and measurement times was found to be statistically significant (F = 7.163; *p* < 0.01). According to this result, it was observed that wrestlers who performed weight loss were exposed to a 0.87% increase in U_SG_ during the dehydration process and regained 0.41% during the rehydration process. This result shows that wrestlers can regain their lost U_SG_ before a high-level competition, although they were exposed to a statistical fluctuation in their U_SG_ (Table 6).

**TABLE 6:** Comparison of fluctuations in wrestlers’ U_SG_ in a high-level competition.

Variables	Groups/Times	n	Beginning of the camp		Weigh-in		Before the competition		Total	F	p
U_SG_ (g*mL^−1^)	WL	21	1.022 ± 0.006		1.031 ± 0.005		1.027 ± 0.004		1.027 ± 0.005	3.147	0.087
			Dehydration %Δ **=** 0.87			Rehydration %Δ = ‒0.41					
	N-WL	10	1.024 ± 0.004		1.025 ± 0.003		1.025 ± 0.005		1.025 ± 0.004		
			Dehydration %Δ **=** 0.01			Rehydration %Δ = 0.04					
	Total	31	1.023 ± 0.005^b^		1.028 ± 0.004^a^		1.026 ± 0.005^b^			Interaction F = 7.163; p < 0.004**	
	F = 7.531; p < 0.003**										

**p < 0.01; ab: Statistical differences between groups were shown with different letters.

## 4 Discussion and conclusion

The main purpose of this research was to determine the fluctuations in hydration markers of wrestlers for a high-level official wrestling competition. In this research, we hypothesized that wrestlers who lost weight for the competition weigh-in before an official wrestling competition could not regain their decreased body composition components and increased urine specific gravity before the competition. It was determined that the wrestlers who lost weight exposed a decrease of 4.02%, 2.50%, 14.62%, and 2.66% in their body weight, FFM, FM, and TBW, and an increase of 0.87% in their U_SG_ during the dehydration process. During the rehydration process, it was determined that they could regain 2.02%, 1.77%, 2.64%, and 1.87% increase in their body weight, FFM, FM, TBM, and 41% decrease in their U_SG_. Our research results support our hypothesis that the wrestlers could not regain the body composition components lost during the dehydration process (between the beginning of the camp and the weigh-in) during the rehydration process (between the weigh-in and before the competition).

According to the classification of the hydration status indexation by Casa et al. (2000), the reference range for percent change in body weight and U_SG_ was reported as well hydrated (+1 to −1 kg and <1.010), minimally dehydrated (−1 to −3 kg and 1.010–1.020), significant dehydration (−3 to −5 kg and 1.021–1.030), and serious dehydration (>5 kg and <1.030), respectively. According to this classification, it was determined in our research that wrestlers who lost weight at the competition weigh-in were significantly dehydrated in body weight changes (4.02%) and extremely dehydrated in U_SG_ (1,031.33 ± 0.4.99). It was observed that wrestlers who did not lose weight were well hydrated in terms of body weight (−0.25%) and significantly dehydrated in terms of U_SG_ (1,024.50 ± 0.3.27). The fact that wrestlers who did not lose weight were significantly dehydrated in terms of U_SG_ may be due to 50% of them keeping their body weight under control or a unit of training they did before weigh-in.

Previous studies have reported that the body weight lost for weigh-in at a competition cannot be regained in the time between weigh-in and competition time (∼17 h) (Buford et al., 2006; Tarnopolsky et al., 1996; Lakicevic et al., 2021). In our research, it was determined that before the competition body weight averages of the wrestlers (87.65 ± 19.60 kg) could not increase to the averages at the beginning of the camp (88.88 ± 19.79 kg). Calorie input and output are important for weight loss. Generally, wrestlers perform weight loss by restricting food and fluids in addition to intense exercise. Body weight loss causes decreases in FM, FFM, and TBW in wrestlers’ bodies. This situation also causes the energy resources needed by the body to decrease. Although wrestlers are free to consume any food and liquid they want after weigh-in, the shrinking stomach during the dehydration process manipulates the ghrelin hormone and they cannot consume more food. Due to wrestlers’ weight loss practices, UWW moved the competition weigh-in to the morning of the competition and tried to prevent weight reduction practices. However, it has been observed that wrestlers continue to perform weight loss practices.

Lakicevic et al. (2021), have reported that reduced TBW with RWL causes dehydration of the kidneys and acute kidney injury, and even recurrent acute kidney injury. They also reported that it may lead to chronic kidney damage in the future. In another study Sagayama et al. (2014), examined body composition fluctuation and found that TBW and FFM decreased significantly between baseline and weight loss (−5.1% ± 1.8%) and returned to baseline values at weight gain. FM decreased significantly between baseline and weight loss (−15.5% ± 12.1%). FM increased again with weight gain but remained lower than at baseline (−10.0% ± 6.5%). In another study Lukic-Sarkanovic et al. (2024), asked wrestlers to reduce their TBW by 5%, and after a week of training without losing weight, they examined the fluctuations in body composition and reported a statistically significant fluctuation in FM and muscle mass levels. When the fluctuations in body composition components were examined in our research, it was determined that before the competition FFM (76.30 ± 12.65 kg-76.50 ± 12.53 kg) and TBW (55.85 ± 9.25 kg-55.99 ± 9.18 kg) averages returned to the beginning of the camp averages, whereas the FM (11.36 ± 7.90^b^–12.39 ± 8.17^a^ kg) averages were not regained. FM remained lower before the competition compared to the beginning of the camp, this is because the wrestlers did not regain all the weight they lost.

Another hydration indicator we examined in our research is the fluctuation in the U_SG_ of wrestlers. It was determined that U_SG_ before the competition was 1.023 ± 0.005, but it increased to 1.028 ± 0.004 at the weigh-in and decreased to 1.026 ± 0.005 before the competition. It was observed that before the competition U_SG_ averages statistically returned to the beginning of the camp averages but could not be reduced to the initial level. When the literature was examined Demirkan et al. (2011), measured U_SG_ 17 and 3 days before the competition, at the weigh-in, and before the competition, and reported that wrestlers exposed fluctuations in U_SG_. In another study Gurses et al. (2018), reported that the judo athletes regained their lost body weight between the weigh-in and before the competition (∼13 h) despite, the increase in U_SG_ could not be reduced. Similarly Guder (2020), examined wrestlers’ U_SG_ between the weigh-in and the before competition (∼2 h) and reported that although the wrestlers had a lower U_SG_ in the competition, they had a U_SG_ above the reference range (≥1.020) and were still dehydrated.

As a result, it was determined that before a high-level official wrestling competition, wrestlers still preferred weight loss practices and could not regain their body weight and FM lost during the dehydration process before the competition. Although it was determined that the wrestlers were exposed to fluctuations in terms of U_SG_ and there was no statistical difference between their U_SG_ at the beginning of the camp and before the competition, it was determined that they could not completely regain their U_SG_.

## 5 Suggestions

In the 2024 Paris Olympic Games, an Indian female wrestler who qualified for the final in 50 kg could not lower her body weight to 52 kg in the final morning (the next morning) weigh-in and was eliminated from the Paris Olympic Games (Basu, 2024). Despite the many tragic events (naked weighing, athletes having their hair cut, heart attacks, and deaths due to dehydration) resulting from the effects of weight loss (Artioli et al., 2010; Litsky, 1997; Villamón et al., 2004), it is seen that wrestlers still practice weight loss practices. According to our research results, the following precautions can be taken into consideration to prevent weight loss practices:

a) Keeping the body weights of wrestlers under control by official UWW official resources even during the off-season and preparation and competition periods,
b) National and international seminars should be organized to educate wrestlers on the effects of hydration status on physical performance,
c) Wrestlers should be informed on how to monitor their hydration status,
d) Instead of RWL practices, ensure that wrestlers perform gradual weight loss practices of less than 1.5% per week,
e) Ensuring that wrestlers perform weight loss practices of less than 5% of their competition weights,
f) Providing that wrestlers are banned from competitions if they are not properly hydrated through hydration markers (e.g., Body fat percentage, or U_SG_),
g) For schoolboys and cadets, the number of weight classes, which is 10, can be increased to 14–15. For the Junior and Senior categories, tolerance in Continental, World, and Olympic Games can be realized with +2 kg tolerance on the first day of the competition weigh-ins, as in UWW’s International Tournaments. In this way, wrestlers’ weight loss can be prevented at a higher level.
h) If the UWW cannot prevent the weight loss practices, new competition weights will be created every 5–10 years with a 3–5 kg increase/decrease.

## 6 Limitations

As with every study, this research has some limitations. One of our most important limitations is that since the athletes were from the competition camp, no diet intervention was applied to the wrestlers. Instead of a controlled weight loss practice by the researchers, the athletes lost their body weight with methods they knew and felt comfortable with (except for the use of diuretics). Of course, this research was only related to senior Greco-Roman wrestlers. There is a need for larger sample studies that examine the hydration status of freestyle and women wrestlers and wrestlers in different age categories and that include other combat athletes.

## Data Availability

The raw data supporting the conclusions of this article will be made available by the authors, without undue reservation.
